# A Virtual Smash Room for Venting Frustration or Just Having Fun: Participatory Design of Virtual Environments in Digitally Reinforced Cancer Rehabilitation

**DOI:** 10.2196/29763

**Published:** 2021-10-07

**Authors:** Johanna Persson, Douglas Clifford, Mattias Wallergård, Ulrika Sandén

**Affiliations:** 1 Department of Design Sciences Lund University Lund Sweden

**Keywords:** virtual reality, virtual environment, cancer rehabilitation, emotions, participatory design, virtual smash room, human factors

## Abstract

**Background:**

Cancer rehabilitation is central for helping patients and relatives create a functional everyday life based on the changes in life conditions. The needs are highly individual and include physical, mental, and social challenges. Cancer rehabilitation programs offer coping strategies, including guidelines on how to handle emotions.

**Objective:**

This paper presents a participatory design activity where patients in cancer rehabilitation use a virtual smash room, which is a virtual environment where the user can break things, mainly porcelain or glass items such as vases or plates. The objective is to understand attitudes to, and some effects of, using this application, as well as eliciting ideas of other virtual environments that would be desired.

**Methods:**

The virtual environment presented here, the virtual smash room, was designed at the request of a patient with cancer who wanted a tool for venting frustration. In this virtual environment, the user can break porcelain, vases, and plates. Patients participating in a week-long cancer rehabilitation program tested the virtual smash room and reported their experiences through a questionnaire. The questionnaire comprised three sections: (1) a subset of the Intrinsic Motivation Inventory (IMI), (2) a subset of the Virtual Reality Symptoms Questionnaire (VRSQ), and (3) a free-text response section.

**Results:**

A total of 101 responses were gathered. The results from the IMI questions showed that the participants found the virtual experience enjoyable (mean 4.52, maximum 5, SD 0.73), and it helped them retain their focus (mean 4.44, maximum 5, SD 0.74). The VRSQ revealed that there were only minor symptoms related to general discomfort (5.9%, n=6), fatigue (5.9%, n=6), nausea (3.0%, n=3), and tired eyes (8.9%, n=9), while several participants experienced dizziness (22.8%, n=23). Since only postmeasurements were gathered, nothing could be concluded about the prevalence of these symptoms before testing. The free-text responses indicated that the user group had many ideas for other virtual environments to use in cancer rehabilitation.

**Conclusions:**

This study presents a concept of using virtual reality in the cancer rehabilitation process and exemplifies activities of patient participation in the design process. Virtual reality has potential in being both distracting and enjoyable, while certain aspects of cybersickness might be especially important to consider for a user group already experiencing physical and mental issues. The results will act as input in the process of further designing virtual applications in digitally reinforced cancer rehabilitation.

## Introduction

The global cancer burden is continuously rising, with more people living with the effects of cancer illness and treatments [1-3]. Both patients and their relatives find it difficult to find a satisfactory and productive life after cancer treatment [4-6]. This is where cancer rehabilitation plays an important role, as patients are helped to return to activities of daily living by overcoming physical, emotional, or social issues affecting their quality of life [7]. The demand for cancer rehabilitation is growing. It is difficult, however, to meet these demands in an already pressured health care system, especially since the effects of both cancer and cancer treatments are highly individualized and can be very complex. They may include physical aspects, such as pain, physical fatigue, and balance issues; mental aspects, such as mental fatigue, distress, and anxiety; social aspects, such as managing relations and adjusting the work situation; and economical aspects owing to a low working capacity. Alternative ways of organizing cancer rehabilitation that take into account this complexity along with the individual needs of each patient need to be addressed [4-6,8]. Digital solutions, including websites, mobile technology, wearables, and virtual reality (VR), are being explored in cancer rehabilitation as a way to empower patients and ease the burden on the health care system [9-11].

In a Swedish research program, the opportunities for digital support in cancer rehabilitation have been explored in a participatory process, in which researchers, patients, patient organizations, health care staff, and their organizations have cocreated ideas and concepts for this purpose. One patient with cancer, and similarly a researcher and coauthor of this paper, expressed a desire for a tool that would distract and enable venting one’s frustration when dealing with specific situations in the cancer rehabilitation process. The use of VR for such a tool was proposed as a potential method, and the idea of a virtual environment where objects could be smashed was devised. This was the background for creating the specific application of a virtual smash room, corresponding to the real-world analogy referred to as “smash rooms,” “rage rooms,” or “anger rooms” [12].

VR enables users to immerse themselves in an alternative reality where they experience presence; that is, the sense of being present in the environment depicted by the VR system [13]. Here, they can interact by reacting to the actions and objects the virtual environment encompasses. Research shows that people react in VR in ways that are similar to how they react to corresponding real-world environments [14,15]. There are also indications that virtual nature experiences can promote recovery from stress [16], and that interacting with virtual scenarios can elicit or strengthen different emotions in a user [17]. A cancer diagnosis inevitably evokes a lot of emotions [4,18-20], including fear, anxiety, frustration, hope, and guilt. Many patients also struggle with existential issues [21]. Life changes in different ways and coping strategies on how to handle this are being explored [22-24]. It is thus worth exploring if VR can be a complement to today’s methods for coping in cancer rehabilitation.

Working participatory in health care, and making the patient an active part of health care interventions, is an approach to let patient’s needs and perspectives guide the design of solutions and the changes to clinical practice [25]. Furthermore, this does not simply involve asking patients what their problems are and then create solutions based on them, but rather this involves continuous activities in which patients and designers define and redefine the problems and iteratively create potential solutions [26].

This study reports on such a participatory design activity where patients in cancer rehabilitation use the virtual smash room, with the objective to understand attitudes to, and some effects of, using this application. Our results would provide further input to the concept of using virtual applications for coping in cancer rehabilitation.

## Methods

### The Virtual Smash Room

The virtual smash room is a virtual environment where the user can break things, mainly porcelain or glass items such as vases or plates. The application was developed in a participatory process in which a key user—the patient, researcher, or coauthor—continuously tested and provided feedback until a VR application was ready to be evaluated by a wider user group [27].

**Figure 1:**
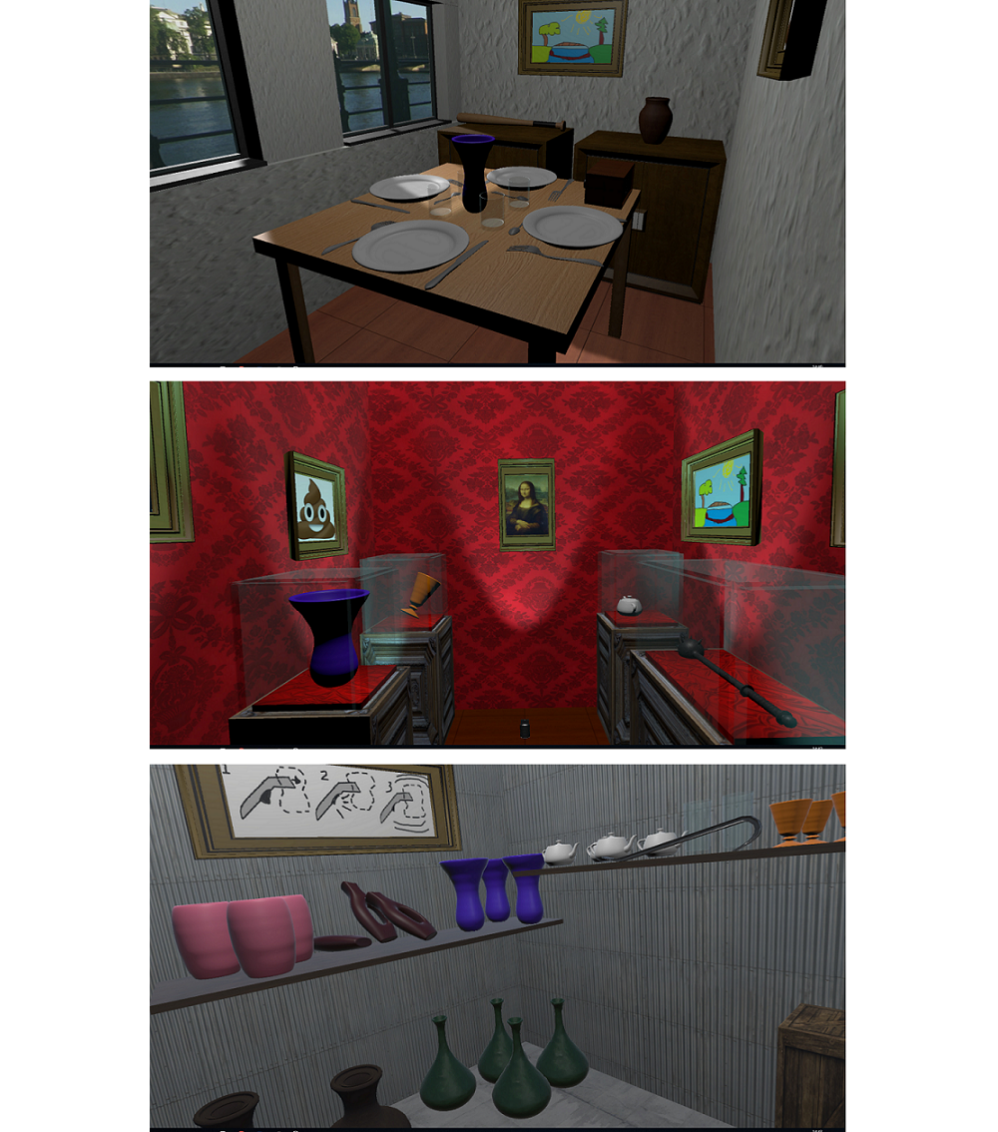
Screenshots from the 3 settings in the virtual smash room (from top to bottom): a dining room with glass and porcelain, a museum with glass showcases, and a factory with large vases and boxes.

The virtual smash room consists of 3 different settings, each with its own theme: a dining room with a table and 2 cupboards filled with glass and porcelain, a museum with glass showcases containing fragile objects, and a factory containing large vases and boxes that require a little more force to break (Figure 1).

The virtual smash room was developed using Unity for usage with an HTC Vive VR system. The latter is a head-mounted display (HMD) accompanied by 2 hand controllers for manual interaction with the displayed environment. The movements of the HMD and the hand controllers are registered by an optical tracking system capable of tracking an area of approximately 3.5 × 3.5 meters. Consequently, the user can move around within a limited area and interact with objects in the virtual environment by using the hand controllers. The user can pick up breakable objects and throw them or smash them against surfaces in the room. There are also virtual tools the user can pick up and use to smash the breakable objects, such as a wooden paddle, a morning star, a hammer, or a crowbar.

**Figure 2:**
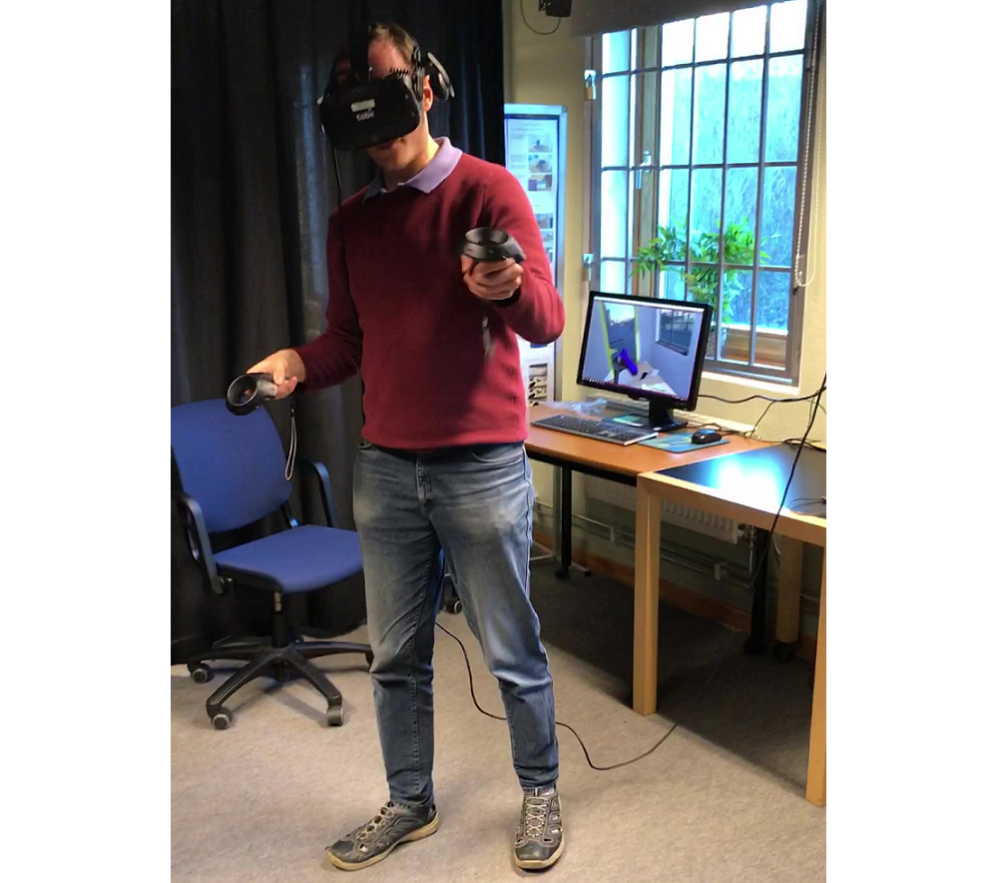
One of the authors using the VR application, wearing the HTC vive with the head-mounted display and the handheld controls. The computer screen in the background shows what the user is seeing.

When used as envisioned, the virtual smash room requires the user to be physically active by moving around and waving his or her arms. The user can interact either by standing up or sitting down if so desired, or required owing to physical limitations. Figure 2 illustrates one of the authors using the virtual smash room.

### Setting

The location of the study was a cancer rehabilitation venue in Sweden. A room was made available to house the virtual reality equipment for patient usage. The venue hosts a week-long cancer rehabilitation program for patients. Each week, up to 16 people participate in activities including the following:

1. Lectures and practical exercises related to physical and psychological side effects, fatigue, emotional effects, etc.
2. Physical exercise and body literacy with a physiotherapist.
3. Mindfulness, yoga, and qigong both indoors and outdoors.
4. Group and individual therapeutic conversations.

The VR experience was scheduled in the middle of the week on the day focused on patient’s emotions during their cancer trajectory.

### Study Design

In the early phase of the participatory design process, ideas and concepts are explored together with user groups to collect input and find ways for further development. Before entering the stage of validating an application for certain effects, it is central to ensure that it is the right application that is being developed. The purpose of testing the virtual smash room in the cancer rehabilitation venue indicated above was to gain input regarding the use of a VR application for ventilating emotions by the actual user group as a formative step in the development process. The focus in the test situation was to capture reactions to the VR application itself, to determine whether it was engaging to use, and to allow for for alternative applications. Accordingly, the virtual smash room served as an artefact for inspiration.

All participants in the rehabilitation week were offered to test the virtual smash room, except for certain weeks when, for example, the group of patients were considered too fragile, or part of the patients needed to spend time on other activities. The staff of the cancer rehabilitation venue determined this from week to week.

A member from the staff was present throughout the whole session. That person introduced the technology and potential side effects, and assisted participants in using the system. The HTC Vive over-ear headphones were used to provide sound feedback but never completely obscured the sound from other people in the room; thus, communication between the participant wearing the HMD and others was possible when using the VR system. Since this is a sensitive user group that might experience physical issues, mental fatigue, and emotional distress, the test was designed to minimize the effort from the participants. Hence, there were no strict instructions, and the participants were free to test a longer or shorter time, and test one or several of the “rooms” in the application. The participants could furthermore choose to either stand or sit down while using the VR system.

The study was performed in line with ethical standards and was approved by the Swedish Ethical Review Authority (2019-01542). Each person who accepted to participate received information about the study and his/her right to end the participation at any time.

### Data Collection

After testing the VR system, each participant was asked to fill in a paper questionnaire to provide data about their experience. The questionnaire comprised three sections based on content from: (1) the Intrinsic Motivation Inventory (IMI) [28,29], (2) the Virtual Reality Symptoms Questionnaire (VRSQ) [30], and (3) a free-text response section.

Since just being in the virtual environment can be a tiring experience in and of itself for this user group (many already experienced mental and physical fatigue), a limited selection of questions to answer was chosen. No personal data, regarding age, gender, or health information, was collected since it was not considered relevant at this stage of the design process. The questionnaire included nine items: 4 from the IMI and 5 from the VRSQ, and 1 section for free-text answers. The process of designing the questionnaire was carried out together with the patient with cancer/researcher/coauthor, and the questions were carefully selected to provide a relevant representation of the content of each questionnaire, without risking that the users did not have energy to answer any questions at all.

The IMI instrument assesses multiple dimensions of a person’s experience of a specified activity, in this case the VR system. The original instrument measures the following: interest/enjoyment, perceived competence, effort, value/usefulness, felt pressure and tension, and perceived choice. The IMI statements can be adjusted so that only certain dimensions are in focus, or can be rephrased to match the specific activity [31]. The 2 dimensions—interest/enjoyment and felt pressure and tension—were considered most important for evaluating the virtual environment for this user group. Only a selection of the questions from these subscales (4 in total) where used at this point to minimize the size of the questionnaire. A 5-point Likert scale was used, ranging from 1=not at all true to 5=very true.

The VRSQ consists of 13 questions that measure physical symptoms, such as headache or nausea that may be experienced when using a VR system [30]. Potential symptoms are important to detect since the user group can be extra sensitive to these effects owing to their disease profile. Again, a subset of questions was selected, to make sure that the questionnaire was not too extensive. Five of the original questions were included to capture any symptoms of general discomfort, fatigue, headache/dizziness, nausea, or tired eyes. The response alternatives were limited to yes/no.

In the free-text section, participants could comment on their experience in their own words. They were also asked to write down ideas they had for other environments they would like to experience in VR.

## Results

### Results Overview

In total, 101 questionnaires were collected from unique participants who tested the virtual smash room at the cancer rehabilitation venue, from October 2019 to March 2020.

Tables 1 and 2 present the results from the IMI and VRSQ parts of the questionnaire. Table 1 lists the 4 IMI statements relating to “interest/enjoyment” and “felt pressure and tension.” The results show that the participants agreed that the VR experience was enjoyable and that it held their attention and were not distracted by other things around them. The results indicate that the majority did not feel tense and were fairly relaxed. Several participants did, however, indicate that they felt tense or did not feel completely relaxed in in the virtual environment. Nonetheless, one participant explicitly stated in the free text that it was a positive tension—something exciting—rather than a negative tension.

**Table 1:** Responses to the 4 statements from the Intrinsic Motivation Inventory. The scale ranged from 1=not at all true to 5=very true.

Statements	Intrinsic Motivation Inventory score, mean (SD)
The virtual experience was enjoyable.	4.52 (0.73)
I was able to maintain my attention while doing this activity.	4.44 (0.74)
I felt tense while doing this activity.	2.27 (1.14)
I felt relaxed while doing this activity.	3.67 (1.11)

Table 2 summarizes the occurrence of various physical symptoms. It shows the percentage and number of people who answered “yes” to the respective questions about sensing the symptom. “Headache or dizziness” is the most prominent problem, with 23 of 101 (22.8%) participants stating that they experienced it. It is worth emphasizing that these symptoms were only measured postquestionnaire, and the extent to which the participants felt headache or dizziness before testing VR or how susceptible they are to these symptoms in general are not known.

**Table 2:** Responses to the 5 questions from the Virtual Reality Symptoms Questionnaire. The table shows the number of participants answering “yes” when asked if they experienced the symptoms.

Symptoms	Participants, n (%)
General discomfort	6 (5.9)
Fatigue	6 (5.9)
Headache or dizziness	23 (22.8)
Nausea	3 (3.0)
Tired eyes	9 (8.9)

### Free-Text Responses

Almost half of the participants (n=49/101) wrote a free-text response. These responses were grouped into six different categories: positive/enjoyable experience (20 entities), physical symptoms (3 entities), difficulties in gripping the virtual tools (7 entities), problems with the hardware (4 entities), using the application together with others (3 entities), and additional suggestions on how to use VR in the cancer process (23 entities). Some of the responses contained information such that they were sorted into several categories, explaining that there are more entities in the 6 categories than the 49 responses.

In total, 20 responses contained comments about the virtual smash room being an enjoyable experience, through statements such as the following: “A lot of fun,” “I would like to do this again,” and “Nice sound when breaking things.” Three participants additionally emphasized their physical symptoms in the free-text section although they had already answered questions about them in the questionnaire. Two participants experienced dizziness and the third one experienced arm fatigue. Seven participants experienced difficulty in gripping the virtual tools used to break things in the virtual smash room. These tools were sometimes referred to as “weapons” as in the following statement: “It was difficult to hold on to the ‘weapons’.” Four users expressed problems with the hardware. One of them wore glasses while wearing the HMD and expressed that this resulted in a blurred view. Two users complained about the handheld controllers temporarily failing and consequently there was a mismatch between the real-world and virtual actions. The fourth user was worried about tripping on the wires.

Three participants commented on using the virtual smash room together with the other participants. Two participants expressed this as a positive experience, while another participant found it to be a negative experience since this activity is about expressing emotions, which can be a very private experience. Finally, 23 additional suggestions on how to use VR in cancer rehabilitation were received; a few were directly related to the virtual smash room, with some participants stating that they wanted to be able to switch to some other room, and other suggestions were of a more general character where a desire to test other environments arose, without specifying exactly which ones. Several participants wanted something calmer, preferably an outdoor nature environment. One participant wanted to organize rather than disorganize: “Instead of smashing things, for example, set the table, arrange flowers, furnish a room, look for things.” Other suggestions were related to sports and hobbies (“Bowling. Darts” and “Go skiing, fishing, hunting”).

## Discussion

### Overview

This study explored the use of VR in cancer rehabilitation. A virtual smash room was developed on request from a patient with cancer or researcher, as a tool for venting frustration, and then evaluated by 101 patients in a cancer rehabilitation program, as part of a participatory design process of digitally reinforced cancer rehabilitation.

### Principal Findings

The majority of the patients who tested the virtual smash room thought it was a positive and enjoyable experience. Nonetheless, several participants felt tense or not completely relaxed, and the prevalence of headache and dizziness indicates that the virtual experience is not comfortable for everyone. However, using only a postmeasure of these symptoms is not enough to state that it is a direct effect of the VR experience. For future testing, a premeasure of the participants’ susceptibility to the measured symptoms is required. Since fatigue or general discomfort might be further prevalent in this user group, it is necessary to thoroughly investigate these effects.

The virtual smash room is an environment in which the user remains almost stationary. He/she can take a few steps in either direction and move the head in any direction, but no more locomotion than that is possible. This implies that all movements in the virtual environment correspond well with those in the real world, which decreases the risk of feeling discomfort, dizziness, or nausea [32]. Even so, despite accurate room-scale tracking, Yildirim [33] reported that cybersickness is still a prevalent human factor issue in modern VR headsets such as HTC Vive and Oculus Rift CV1. Little is known about the underlying reasons, but anecdotal evidence from the VR gaming community suggests the involvement of the so-called screen door effect (SDE). Since the user’s eyes are very close to the display, the area of unlit space between pixels creates a sensation of having your vision disrupted by a black grid or a screen door. The SDE is a common problem in many modern VR headsets, especially those equipped with organic light-emitting diode displays such as HTC Vive. It is, however, important to point out that HTC Vive belongs to the first generation of modern consumer VR headsets, and that the fast-paced technological development will result in increasingly advanced and comfortable VR headsets. For example, the HP Reverb G2 VR headset has a resolution of 2160 × 2160 pixels per eye (as opposed to 1080 × 1200 pixels per eye for HTC Vive), which renders the SDE almost unnoticeable. Another plausible cause of the reported headache/dizziness is related to the fact that the user group likely has a greater tendency to experience these symptoms. Many factors affect one’s susceptibility to cybersickness [34], and this is definitely something to consider and investigate further when developing VR for this user group.

The two dominant free-text answer categories contained (1) comments about the virtual smash room being an enjoyable experience and (2) suggestions for additional virtual environments to use in cancer rehabilitation. These support further exploration of VR in cancer rehabilitation. The fact that several participants complained about difficulties holding on to, or gripping, the virtual objects indicates that the user interface needs redesigning, and possibly also that the hand controllers should be replaced. Tracking of hand gestures or eye gaze, or a combination of these, as proposed by Pfeuffer et al [35], might be interesting alternatives. Even though only 7 of our 101 participants complained about this, it is enough to call for an improvement. This shows that including the primary users in the design process is a central source of information about which applications to develop and which user interface mechanisms to improve.

The participatory approach is worth highlighting in a domain that traditionally has been technology-centered rather than human-centered with regard to the development of new applications [36,37]. This is also relevant from a cancer rehabilitation perspective, since many studies show that being able to influence and participate in one’s own care process is beneficial [38-41]. By participating in the development and implementation of VR in cancer rehabilitation, patients are able to try new ways of experiencing different realities. Through this, we hope that they will be inspired to think about how their own rehabilitation, as well as that for future patients, can benefit from using VR technology.

In the free-text responses of the evaluation, there was an explicit request for nature and relaxing environments, and this is an area where VR can be a complement to real outdoor, natural environments [16,42]. The effect of VR on emotions and the mental state is simultaneously being studied in dementia and geriatric care where applications for reducing anxiety and apathy are explored [43-45]. Learnings from these studies will also be applicable to cancer rehabilitation to some extent.

Other environments and activities that can be explored in VR are desired, whether they are more peaceful or more active. In particular, aspects related to human factors must be considered when implementing VR. This implies that the technical solution is part of a comprehensive system consisting of people, a physical environment, technical artefacts, and a work organization, all of which must function together [46]. In this case, the technical solution includes the virtual environment and the user interface as well as the hardware, the physical surrounding, the conditions of the users, and the organization of the cancer rehabilitation venue. The HMD is still a cumbersome device to handle and wear. The user group also has a higher risk for infection; hence, the hardware needs to be sterilized between use. The environment must accommodate users with physical disabilities; some may need to sit down, and certain virtual environments may be more prone than others to cause dizziness or nausea. If the aim is to manage difficult emotions, simply being in the virtual environment might be so exhaustive that resources, such as a psychologist, should be available for consultation at some stage in the experience.

### Limitations

The original idea came from experiencing frustration with certain issues in the cancer rehabilitation process, and the ideal solution would be to offer the virtual smash room in relation to such a situation, when a person feels frustrated. In this study, the application was presented as a test activity at 1 specific moment during the rehabilitation week, and not at a moment when the participants necessarily felt frustrated, which makes it difficult to draw conclusions about the VR application’s ability to be a tool for venting frustration or for coping in general. It did, however, evoke feelings of having fun, which is an important aspect in the process of handling frustration and stress [47]. If the participants had had on-demand access to VR equipment and could use it whenever they wanted, in private or in pairs, and could choose a VR application that matched their current state of emotion, perhaps other behaviors would have been observed.

This study is a step in an exploratory phase of the design process; hence, the generalizability of our results is limited. The value of the study is to explore the use of VR as a supplement in cancer rehabilitation together with the specific user group and elicit users’ voices in the development of digitally reinforced cancer rehabilitation. In this analysis, no specific instructions were provided to the user when testing the application. Users were free to test the application for a long or short period in one or several of the “rooms” in the application. It would have been valuable to observe this more thoroughly; for example, to observe what the users did and measure the time during which they interacted with the VR application, but this could not be done owing to practical reasons. The researchers were not allowed at the venue when the patients were there owing to infection risks, and there were no additional staff members who would spend time in performing this activity. This is, of course, challenging for future studies but could be assessed in another setting or with observations using digital tools or video recordings.

### Conclusions and Future Prospects

This study explores the use of VR in cancer rehabilitation, with a virtual smash room designed to evoke feelings, and demonstrates how the patients can be the innovators and participates in the development. The results show that the participants found the VR experience enjoyable and that it distracted them from their surroundings. Some participants experienced dizziness and had problems with the user interface. The user group expressed many ideas for other virtual environments to use in cancer rehabilitation. Our results would serve as input in the process of designing other VR applications for cancer rehabilitation, in participation with the patients, their families, and the staff.

The next step in this process involves broadening the sources of VR experiences to test—either in virtual worlds, augmented worlds, or interactive 360 videos—and explore the surrounding practical aspects of employing this technology with lessons from this feasibility study in mind. Future studies also involve more controlled test setups, including, for example, pre- and postmeasures of symptoms, observations of the time spent in the virtual environment, and individual behavior and attitudes.

## Abbreviations

HMD: head-mounted display
IMI: Intrinsic Motivation Inventory
SDE: screen door effect
VR: virtual reality
VRSQ: Virtual Reality Symptoms Questionnaire
